# Supplementary material for: Food Practice Lifestyles: Identification and Implications for Energy Sustainability
Source: Int J Environ Res Public Health. 2022 May 5;19(9):5638. doi: 10.3390/ijerph19095638 (PMC9103468; doi:10.3390/ijerph19095638)
Supplement: Supplementary file 1 [file ijerph-19-05638-s001.zip › ijerph-1613476-supplementary.pdf]

Supplementary A

Sample Characteristics

The ATUS is designed to be nationally representative of the civilian non-institutional population ages 15 and older and various subgroups [59]. We produced summary statistics using the Eating and Health Module probability weights provided in the ATUS data set. As shown in Table A1, the mean age of our sample was 45 years, with almost two-thirds of the sample in the 25–64 age group (65%), and the remainder divided between the youngest group of 15–24 years (17%) and the oldest group age 65+ (18%). Women made up slightly over half of the sample (52%). The majority of our sample was White (81%) and most respondents were not Hispanic (84%). Less than one-third of respondents had earned a BA (31%), and approximately one-quarter had some college/AA (25%) or a high school diploma/GED (28%), while 16 percent had less than a high school diploma. Most respondents were employed (62%), married (52%) and lived in households of two or less adults (75%). Over one-third of respondents had children under 18 years living in the household (38%), while a much smaller proportion (13%) reported the presence of children under 5.

Almost three-quarters of respondents reported owning their house with a mortgage (72%), while over one-quarter reported renting (27%). Most respondents (54%) reported family incomes higher than $50,000, with the remaining respondents divided between those with income <$25,000 (22%) and income between $25,000 and $50,000 (23%). Over one-quarter of the sample (32%) has household resources that are less than 185% of the estimated official poverty threshold given household size and composition. The vast majority reported having enough food to eat (94%). The largest share of respondents reported living in the South (38%), followed by the Midwest (23%), West (21%) and Northeast (17%). Most respondents lived in Metro areas (84%). The weighted sample is evenly distributed with respect to the diary day of the week (Monday through Friday) and season; results not shown.

Table S1. Respondent and household characteristics (summary statistics).

Variable	Description/Category	Sample Share (%) [1]
Age	15–24	16.8
	34–64	65.0
	65+	18.2
Sex	Male	48.3
	Female	51.7
Race	White	80.9
	Black	12.3
	Asian	4.3
	NA/PI/HI	1.1
	Multiple	1.4
Ethnicity	Hispanic	15.9
	Not Hispanic	84.1
Education	HS or less	44.1
	Some coll/AA	25.4
	BA and higher	30.5
Employment	Employed	61.7

	Unemployed	5.05
	Not in labor force	38.3
Marital Status	Married	52.1
	Widowed	5.6
	Div/sep	11.4
	Never married	30.9
Household size	1–2	75.3
	3–5	14.3
	6+	9.9
Children < age 5 in HH	No child(ren) < 5	86.6
	Child(ren) < 5	13.4
Home Ownership	Owned	72.7
	Rented	27.3
Household Income	<25 K	23.1
	25 K–49 K	22.4
	50 K–99 K	27.1
	100 K+	27.3
Income <185% poverty threshold	<185% poverty threshold	65.2
	>=185% poverty threshold	32.2
	Ref/DK/NIU	2.6
Food Security	Enough food	94.0
	Not enough food	5.4
	Refused/DK/NIU	0.5
Region	Northeast	17.4
	Midwest	23.4
	South	38.0
	West	21.2
Metro Status	Metro	84.0
	Nonmetro	15.2
	Metro NA	0.8

[1] Sample = 16,100; weighted population size = 199,628,063,772.

Table S2. Activity-level Variables.

Analytic Variable	IPUMS Variable Name and ATUS Survey Question
Preparation	ACTIVITY = Food and drink preparation
Eating/Drinking	ACTIVITY = Primary: Eating and drinking; eating and drinking as a part of job<br>ACTIVITY = Secondary: Eating while engaged in other primary activity
Cleaning	ACTIVITY = Kitchen and food cleanup
Groceries	ACTIVITY = Grocery shopping
Purchase	ACTIVITY = Purchasing food (not groceries)
Travel	ACTIVITY = Travel associated with eating and drinking
Activity Duration	ACTIVITY = Minutes
Activity Start	START: HH:MM AM/PM
Activity Stop	STOP: HH:MM AM/PM
Activity Location	WHERE: Home or yard;<br>Away from home or yard

Table S3. Individual and Household Characteristics.

Analytic Variable	Analytic Categories	IPUMS Variable Name, Label and Format
Age	Young adult: 15–24<br>Working-age adult: 25–64<br>Older adult: 65+	AGE: Age (continuous)
Sex	Male	SEX: Sex (2 categories)

	Female<br>DK, refused	
Race	White<br>Black<br>Asian<br>Native American, Pacific Islander or Hawaiian<br>Multiple	RACE: Race (35 categories)
Ethnicity	Hispanic<br>Not Hispanic	HISPAN: Hispanic origin (13 categories)
Education	HS diploma or less<br>Some college/AA<br>BA and higher	EDUC: Highest level of school completed (17 categories)
Employment	Employed<br>Unemployed or not in labor force	EMPSTAT: labor force status (5 categories)
Marital Status	Married<br>Not married	MARST: Marital status (6 categories)
Household size	1–2<br>3–5<br>6+	HH_SIZE: Number of people in household (16 categories)
Children < age 5 in HH	No child(ren) < 5 years<br>Child(ren) < 5 years	AGEYCHILD: Age of youngest household child (17 categories)
Home Ownership	Owned<br>Rented	HHTENURE: Living quarters owned, rented or occupied without rent (3 categories)
Family Income	<$25,000<br>$25,000–<$50,000<br>$50,000–<$100,000<br>$100,000+	FAMINC: Family income (18 categories)
Near-poverty status	Income >= 185% poverty<br>Income < 185% poverty<br>Refused/Don't know/not in universe	POV185: Household income greater or less than 185% of poverty level (5 categories)
Fast-food purchase	Purchased fast food<br>Did not purchase fast food<br>Refused/Don't know/not in universe	FASTFD: Purchased any prepared food from a deli, carry-out, delivery food, or fast food
Metro Status	Metro<br>Nonmetro<br>Metro NA	METRO: Metropolitan/central city status (5 categories)

Supplementary B

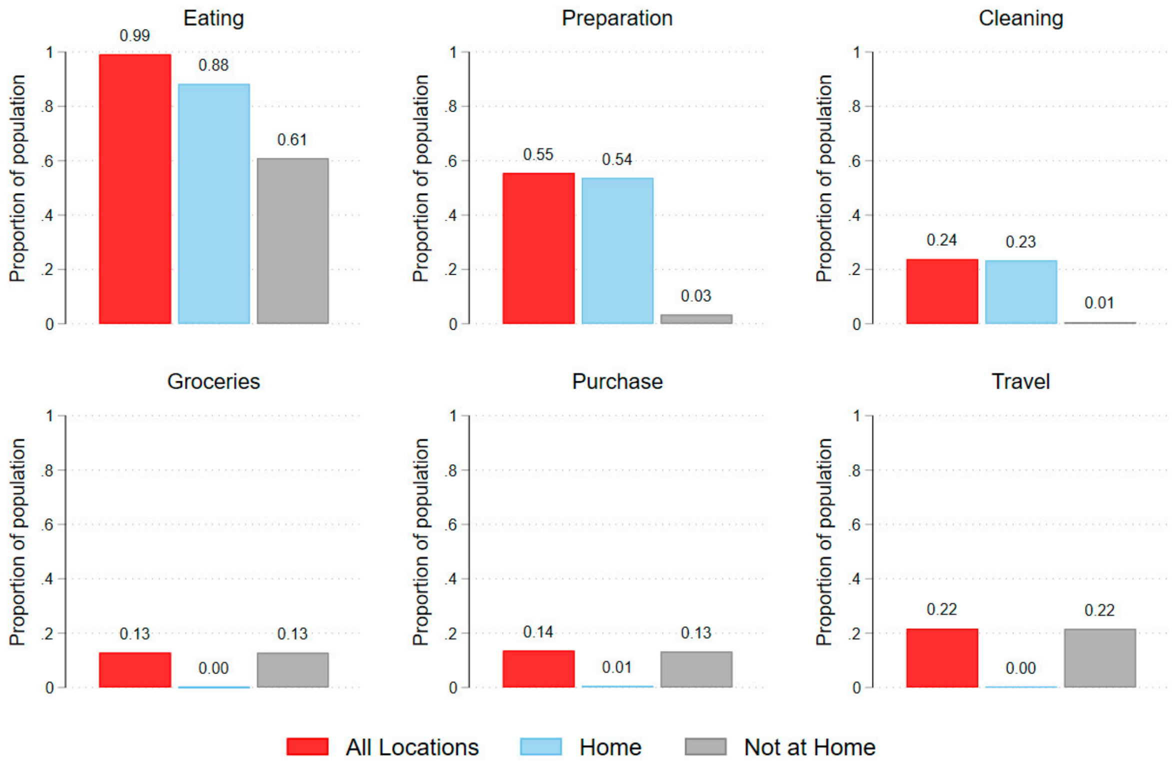

Figure S1. Proportion of population engaging in food-related activities.

Supplementary C

Table S4. Activity duration (number of minutes), conditional on engaging in activity.

Variable	All Locations		At Home		Not at Home	
	Mean	Se	Mean	Se	Mean	Se
Eating/drinking	79.1	0.4	53.1	0.2	51.8	0.8
Primary eating	65.1	0.2	47.8	0.1	48.3	0.3
Secondary eating	30.0	1.0	23.7	0.6	30.0	2.5
Preparation	50.0	0.3	49.5	0.3	34.8	5.8
Cleaning	33.4	0.4	33.3	0.3	28.8	29.3
Grocery shopping	43.2	0.6	46.9	31.7	43.2	0.6
Purchasing food	10.6	0.1	7.3	0.6	10.6	0.0
Food-related traveling	25.1	0.4	14.8	5.3	25.0	0.4

n = 16,100; probability weights applied.

Table S5. Activity duration (number of minutes), not conditional on engaging in activity.

Variable	All Locations		At Home		Not at Home	
	Mean	Sd	Mean	Sd	Mean	Sd
Eating/drinking	78.4	0.4	46.8	0.2	31.6	0.4
Primary eating/drinking	62.1	0.2	39.5	0.1	22.5	0.1
Secondary eating	16.3	0.3	7.3	0.1	9.0	0.2
Preparation	27.7	0.2	26.5	0.2	1.2	0.0
Cleaning	7.9	0.0	7.8	0.0	0.2	0.0
Grocery shopping	5.5	0.0	0.0	0.0	5.5	0.0

Purchasing food	1.4	0.0	0.0	0.0	5.5	0.0
Food-related traveling	5.4	0.0	0.0	0.0	5.4	0.0

n = 16,100; probability weights applied.

Supplementary D

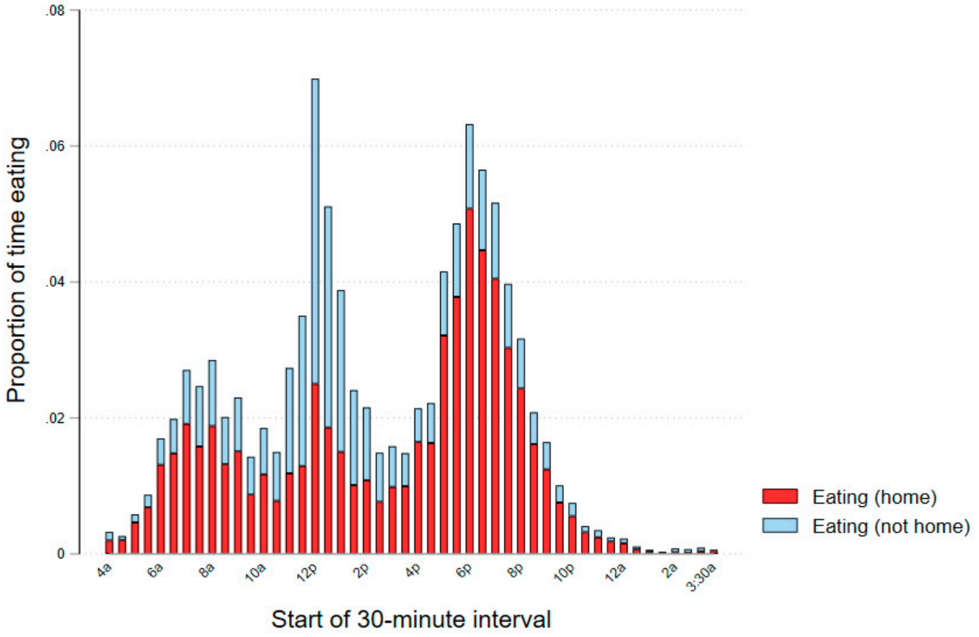

Figure S2. Average time eating by interval and location (as proportion of all eating).

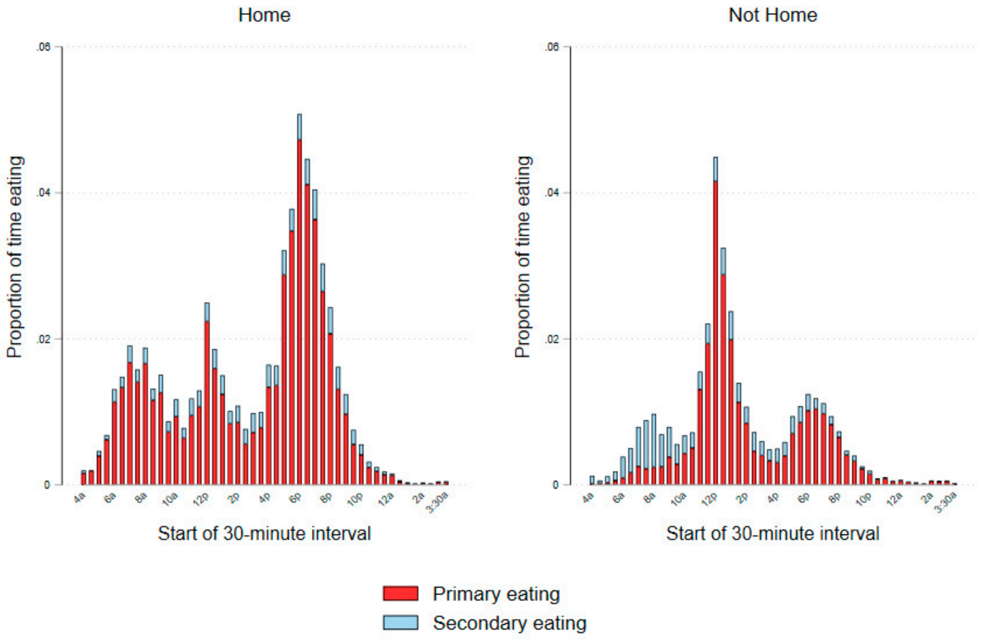

Figure S3. Time spent in primary and second eating by interval and location (as proportion of all eating).

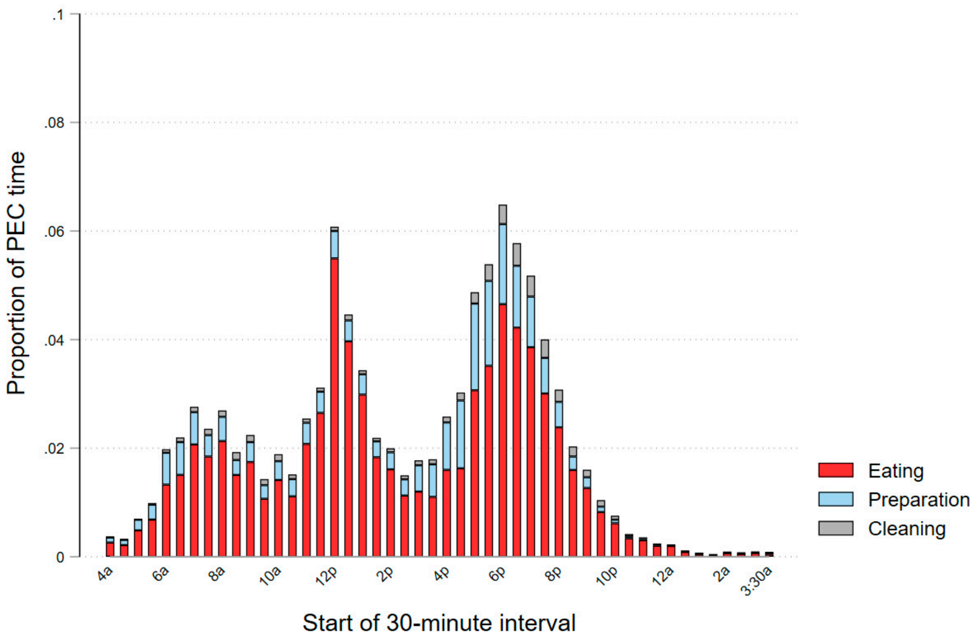

Figure S4. Time spent in food preparation, eating and cleanup by interval (as proportion of all eating).

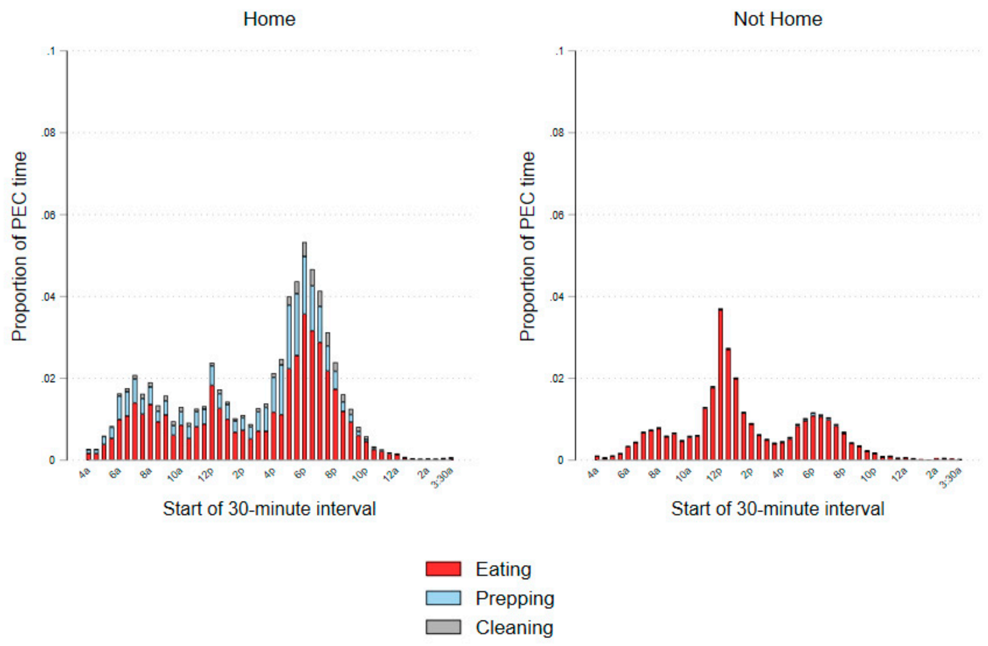

Figure S5. Time spent in preparation, eating and cleaning by interval and location (as proportion of all eating).

Supplementary E

Table S6. Full results from multivariate linear regression panel analysis predicting home eating.

	(1) At-Home Eating	
	**Coeff**	***p*-Value**
Midday (10 a–4 p)	0.0018	(0.529)

Early evening (4 p–9 p)	0.037 ***	(0.000)
Night (9 p–5 a)	−0.032 ***	(0.000)
Current Preparation (current interval)	0.11 ***	(0.000)
Current Cleaning (current interval)	0.040 **	(0.007)
Lag 1 Preparation (1 interval prior to current)	0.27 ***	(0.000)
Lead 1 Cleaning (1 interval after current)	0.052 ***	(0.000)
Lag 2 Preparation (2 intervals prior to current)	0.21 ***	(0.000)
Lead 2 Cleaning (2 intervals after current)	−0.020	(0.189)
Constant	0.044 ***	(0.000)

Fixed-effects model; statistical significance: * $p < 0.05$, ** $p < 0.01$, and *** $p < 0.001$; R-squares: within: 0.11/between: 0.07/overall: 0.10; Sigma: 0.23/Sigma_e: 0.22/Sigma_u: 0.05.

Supplementary F

K-Means Cluster Analysis

K-means cluster analysis is a non-parametric method of identifying groups of observations that are similar on the basis of their responses to multiple variables; it works by classifying observations into *k* groups by minimizing within-cluster variances. We ran the cluster analysis in Stata 15.1 using a squared Euclidean distance algorithm. We performed the cluster analysis on normalized eating duration data over 41 overlapping intervals (of 4 h each), conducting the analyses separately for at-home and not-home activities. (For both locations, the durations were normalized by the total amount of time spent eating during the day for the purposes of comparison.) We used overlapping intervals for the cluster analysis to account for the likelihood that some activities may occur over the course of multiple periods. We chose to conduct the analyses separately by location to allow for potential of distinct at-home and not-home eating patterns.

We ran each K-means cluster analysis for $k = 1$ to 30. Tables S4 and S5 display the scree plots from the cluster analyses. As shown, the scree plots show that both cluster analyses yielded a decreasing Within-Sum-of-Squares (WSS), or sum of squared errors for each 1-unit increase in the number of groups *k*. The change in the WSS decreases with each increase in *k*, especially where $3 < k < 5$. However, neither analysis yielded a clear "elbow" that could be used to identify the ideal number of clusters. We ultimately chose three of each type—at-home and not-home—the results of which were combined into nine unique groups.

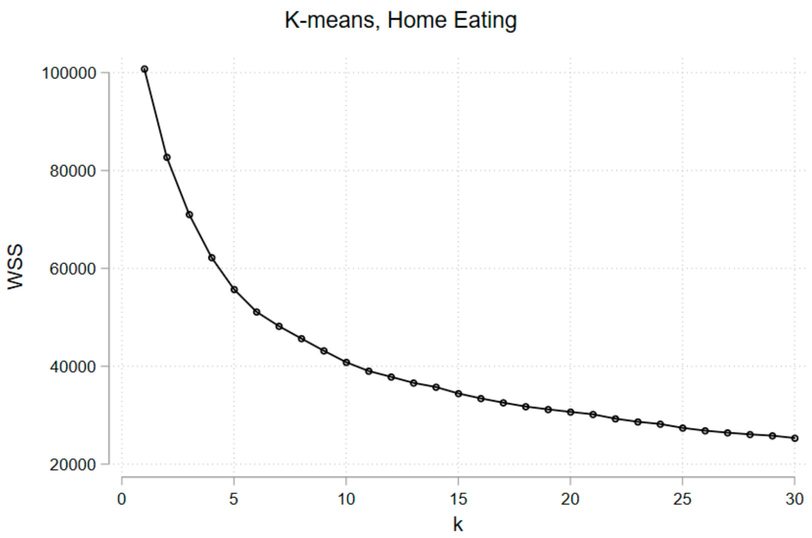

Figure S6. Scree plot from cluster analysis of home eating (as a proportion of all eating).

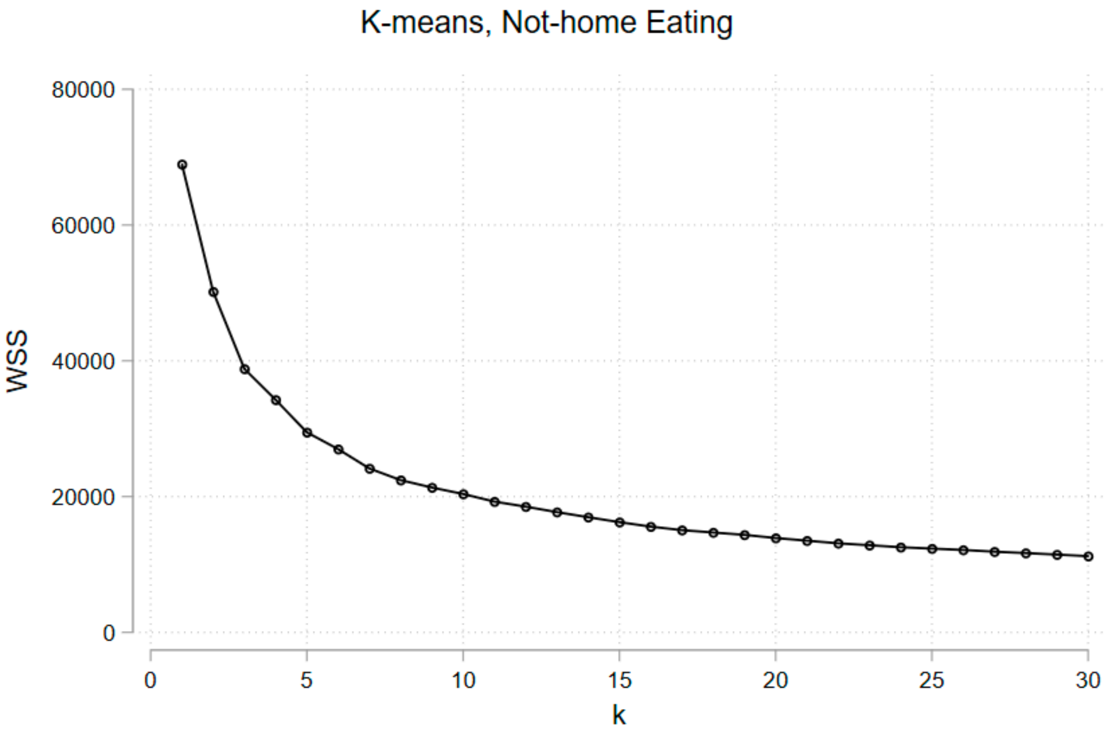

Figure S7. Scree plot from cluster analysis of not-home eating (as a proportion of total eating).

Supplementary G

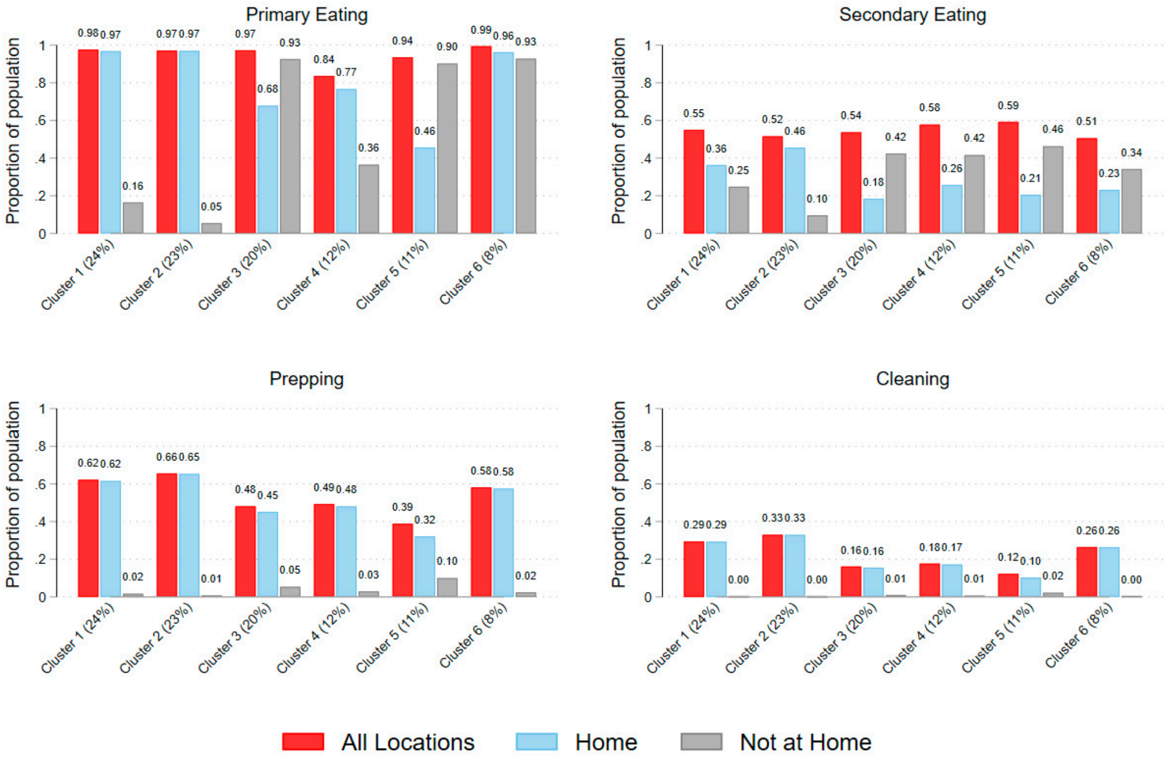

Figure S8. Participation in food-related activities by cluster and location (proportion of cluster).

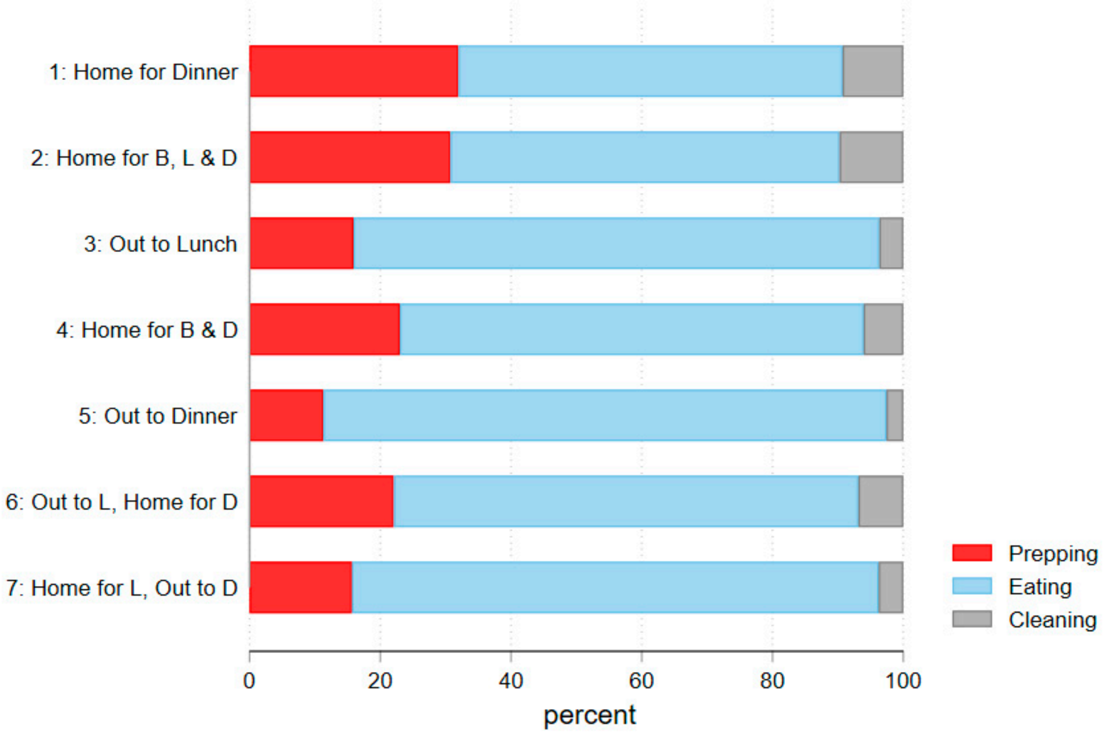

Figure S9. Percent of time preparation, eating and cleaning (as proportion of all time in these activities).

Table S7. Conditional activity duration (number of minutes).

		1: Home D		2: Home B, L and D		3: Out L		4: Home B and D		5: Out D		6: Out L, Home D	
		**Mean**	**Se**	**Mean**	**Se**	**Mean**	**Se**	**Mean**	**Se**	**Mean**	**Se**	**Mean**	**Se**
All	Eating	64.9	0.2	78.1	0.1	89.3	1.9	77.4	0.9	98.7	2.7	74.0	0.3
	Preparation	56.7	0.2	61.5	0.1	36.7	1.9	47.1	0.9	33.3	2.7	39.5	0.3
	Cleaning	34.9	0.2	38.8	0.1	25.1	1.9	34.6	0.9	24.0	2.7	27.0	0.3
Home	Eating	57.8	0.2	75.7	0.1	31.0	1.9	52.2	0.9	25.6	2.7	38.7	0.3
	Preparation	56.5	0.2	61.4	0.1	35.2	1.9	45.3	0.9	28.4	2.7	39.3	0.3
	Cleaning	34.9	0.2	38.7	0.1	25.0	1.9	33.0	0.9	22.4	2.7	26.9	0.3
Not Home	Eating	19.3	0.2	16.8	0.1	66.1	1.9	41.8	0.9	84.2	2.7	35.3	0.3
	Preparation	32.1	0.2	28.2	0.1	32.8	1.9	48.6	0.9	38.3	2.7	15.1	0.3
	Cleaning	12.5	0.2	35.4	0.1	17.3	1.9	63.1	0.9	28.9	2.7	14.2	0.3

Supplementary H

Table S8. Multivariate logistic regression predicting cluster membership.

	(1) Home Dinner		(2) Home B, L and D		(3) Out Lunch		(4) Home B and D		(5) Out Dinner		(6) Out L, Home D	
	**Odds Ratio**	***p*-Value**	**Odds Ratio**	***p*-Value**	**Odds Ratio**	***p*-Value**	**Odds Ratio**	***p*-Value**	**Odds Ratio**	***p*-Value**	**Odds Ratio**	***p*-Value**
Age 15–24 (vs. 25–64)	0.99	(0.919)	0.73 **	(0.002)	1.07	(0.427)	0.71 **	(0.004)	1.84 ***	(0.000)	1.29 *	(0.044)
Age 65+ (vs. 25–64)	0.77 ***	(0.000)	1.70 ***	(0.000)	0.79 **	(0.002)	0.87	(0.118)	0.94	(0.540)	0.59 ***	(0.000)
Female (vs. Male)	1.09	(0.073)	1.17 **	(0.001)	0.90 *	(0.045)	0.71 ***	(0.000)	1.09	(0.201)	1.02	(0.792)
White (vs. Non-White)	1.06	(0.341)	1.07	(0.300)	0.88	(0.051)	0.78 ***	(0.001)	1.10	(0.304)	1.23 *	(0.049)
Not Hispanic (vs. Hispanic)	0.77 ***	(0.000)	1.12	(0.141)	1.52 ***	(0.000)	0.96	(0.648)	0.71 **	(0.001)	0.97	(0.771)
Married (vs. Not married)	1.37 ***	(0.000)	1.11 *	(0.042)	0.82 ***	(0.000)	0.72 ***	(0.000)	0.74 ***	(0.000)	1.43 ***	(0.000)
Child < 5 in HH (vs. No child < 5)	1.04	(0.536)	1.14	(0.075)	0.89	(0.102)	0.97	(0.718)	0.77 **	(0.009)	1.26 *	(0.011)
BA and higher (vs. No BA)	0.90	(0.051)	1.04	(0.501)	1.02	(0.696)	0.93	(0.276)	1.19 *	(0.020)	1.01	(0.871)
Employed (vs. Not empld)	0.65 ***	(0.000)	0.31 ***	(0.000)	2.78 ***	(0.000)	1.30 ***	(0.001)	1.68 ***	(0.000)	3.13 ***	(0.000)
Own house (vs. Not owned)	0.97	(0.617)	1.00	(0.967)	0.96	(0.535)	0.97	(0.631)	1.04	(0.602)	1.19	(0.073)
<=185% poverty (vs. >185% pov)	1.01	(0.840)	1.39 ***	(0.000)	0.75 ***	(0.000)	1.02	(0.823)	0.87	(0.128)	0.86	(0.122)
Fast food (vs. No fast food)	0.84 ***	(0.000)	0.84 ***	(0.001)	1.21 ***	(0.000)	0.98	(0.720)	1.43 ***	(0.000)	0.99	(0.914)
Nonmetro (vs. Metro)	0.92	(0.228)	1.06	(0.372)	0.99	(0.887)	1.12	(0.186)	0.92	(0.349)	1.06	(0.571)
Observations	15,872		15,872		15,872		15,872		15,872		15,872	

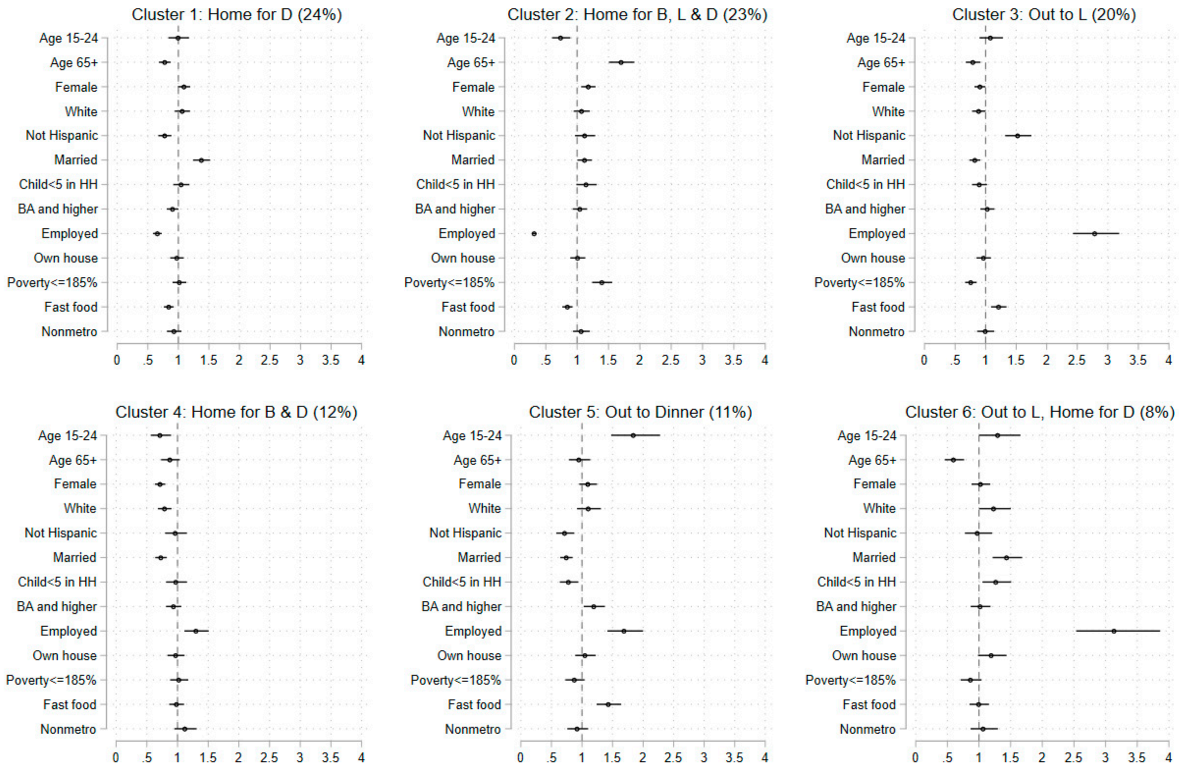

Figure S10. Multivariate logistic regression predicting cluster membership (odds ratios).
